# Dynamical properties of elemental metabolism distinguish attention deficit hyperactivity disorder from autism spectrum disorder

**DOI:** 10.1038/s41398-019-0567-6

**Published:** 2019-09-25

**Authors:** Christine Austin, Paul Curtin, Austen Curtin, Chris Gennings, Manish Arora, Kristiina Tammimies, Johan Isaksson, Charlotte Willfors, Sven Bölte

**Affiliations:** 1 Department of Environmental Medicine and Public Health, Icahn School of Medicine at Mount Sinai, One Gustave L Levy Place, Box 1057, New York, NY 10029 USA; 2 Karolinska Institutet Center of Neurodevelopmental Disorders (KIND), Centre for Psychiatry Research, Department of Women’s and Children’s Health, Karolinska Institutet, & Stockholm Health Care Services, Stockholm County Council, Stockholm, Sweden; 3 Department of Neuroscience, Child and Adolescent Psychiatry Unit, Uppsala University, Uppsala, Sweden; 4 Department of Molecular Medicine and Surgery, Center for Molecular Medicine, Karolinska Institutet, Stockholm, Sweden; 5 Curtin Autism Research Group, Essential Partner Autism CRC, School of Occupational Therapy, Social Work and Speech Pathology, Curtin University, Perth, WA Australia

**Keywords:** Predictive markers, ADHD, Autism spectrum disorders

## Abstract

Attention deficit hyperactivity disorder (ADHD) and autism spectrum disorder (ASD) are neurodevelopmental conditions of overlapping etiologies and phenotypes. For ASD, we recently reported altered elemental metabolic patterns in the form of short and irregular zinc and copper cycles. Here, we extend the application of these biomarkers of prenatal and early postnatal elemental metabolism to distinguish between individuals diagnosed with ADHD and/or ASD and neurotypical controls. We recruited twins discordant for ADHD, ASD and other neurodevelopmental diagnoses from national twin studies in Sweden (*N* = 74) diagnosed according to DSM-5 clinical consensus and standardized psychiatric instruments. Detailed temporal profiles of exposure to 10 metals over the prenatal and early childhood periods were measured using tooth biomarkers. We used recurrence quantification analysis (RQA) to characterize properties of cyclical metabolic patterns of these metals. Regularity (determinism) and complexity (entropy) of elemental cycles was consistently reduced in ADHD for cobalt, lead, and vanadium (determinism: cobalt, β = −0.03, *P* = 0.017; lead, β = −0.03, *P* = 0.016; and vanadium, β = −0.03, P = 0.01. Entropy: cobalt, β = −0.13, *P* = 0.017; lead, β = −0.18, *P* = 0.016; and vanadium, β = −0.15, *P* = 0.008). Further, we found elemental pathways and dynamical features specific to ADHD vs ASD, and unique characteristics associated with ADHD/ASD combined presentation. Dysregulation of cyclical processes in elemental metabolism during prenatal and early postnatal development not only encompasses pathways shared by ADHD and ASD, but also comprise features specific to either condition.

## Introduction

Attention deficit hyperactivity disorder (ADHD) is a common neurodevelopmental condition affecting an estimated 5.3% of children and 2.5% of adults worldwide^1,2^. Heritability estimates average 75% with a polygenic liability comprising both common and rare variants, suggesting that environmental exposures are also at play^3–5^. However, the role of specific environmental exposures and their metabolism in the etiology of ADHD and co-occurring neurodevelopmental conditions, such as autism spectrum disorder (ASD)^6^, remains unclear^7^. Few studies have investigated the association between elemental exposures and ADHD (for reviews, see^8^ and^3^). In addition, those studies have exclusively relied on cross-sectional measures of exposure, primarily blood metal concentrations. They found differences between ADHD and non-ADHD controls for lead, mercury, manganese, iron, zinc, and copper^9–18^. Unfortunately, this research does not include information on the timing of exposures relative to their effects on neurodevelopment, or permit contrasts between different neurodevelopmental disorders. For example, lead is the most widely investigated toxic exposure linked to ADHD, but study findings derived from a single time-point measurement do not resolve temporal variations in exposure during critical developmental windows^3^. Using novel tooth matrix biomarkers, we recently demonstrated that essential and toxic elements are differentially regulated in ASD^19^. This was achieved by focusing analyses on time variant cycles in elemental metabolism, which would be missed in analyses relying solely on single time-point concentrations. The combination of multiple cohorts in that study, each drawn from different sites with varying enrollment protocols, nonetheless did not allow a fine scale differentiation of ASD and ADHD phenotypes, as were characterized in the RATSS cohort.

Here, we test the hypothesis that cycles of essential and toxic elements are dysregulated during prenatal and early postnatal development in ADHD and that these profiles distinguish individuals with ADHD from individuals with ASD as well as ADHD/ASD combined presentation^6^. We utilize recurrence quantification analysis (RQA)^20–22^, a non-linear method of characterizing signal dynamics, particularly the frequency, duration and complexity of cyclical processes in longitudinal time-series measures of elemental metabolism. In a well-characterized population-derived sample of twins in Sweden^19,23,24^, which includes monozygotic twins discordant for ADHD, we apply RQA to fine scale temporal profiles of metal uptake during the prenatal and early postnatal period made possible by recently developed tooth matrix biomarkers. The twin design allows us to account for underlying genetic factors, especially in monozygotic twins.

## Materials and methods

### Participant characteristics

Study samples were pulled from the Roots of Autism and ADHD Twin Study in Sweden (RATSS)^23^. Participant recruitment and tooth collection within RATSS have been previously described^24^. Briefly, we collected and analyzed teeth from 74 participants: 30 complete twin pairs, 1 triplet group, and 11 individuals from twin pairs whose sibling did not donate a tooth. For our primary analysis, we measured metals in 13 cases of ADHD, 8 ASD, 12 ADHD/ASD combined, and 41 neurotypical controls (see Table 1 for participant characteristics). This sample size accounts for ~50% of the whole RATSS cohort of tooth shedding age. The clinical aspect of the study and sample collection were approved by the Swedish National Ethical Review Board. All participants gave informed consent. Analyses were also approved by the Institutional Review Board of the Icahn School of Medicine.

**Table 1:** RATSS Participant Characteristics. (A) Participant numbers, gestational age and birth weight. (B) Mean IQ (standrd deviation) by diagnostic category

A							
	N	ADHD	ASD	ADHD/ASD comorbid	Typically developing	Mean gestational days (SD)	Mean birth weight (Kg) (SD)
Male							
Overall	45	7	6	8	24	247 (24)	2.4 (0.7)
MZ	23	3	5	3	12	239 (13)	2.5 (0.5)
DZ	18	3	1	4	10	257 (16)	2.6 (0.6)
Singleton	4	1	0	1	2	230 (55)	2.1 (0.1)
Female							
Overall	29	6	2	4	17	246 (24)	2.4 (0.7)
MZ	10	0	1	4	5	257 (10)	2.5 (0.6)
DZ	13	6	1	0	6	246 (37)	2.2 (0.1)
Singleton	6	0	0	0	6	259 (18)	2.4 (0.4)

B	
Group	Mean IQ (SD)
ADHD	101.15 (4.16)
ASD	86.12 (20.97)
ADHD/ASD comorbid	90.75 (19.75)
Typically developing	99.85 (15.18)

### Clinical assessment

Participants were diagnosed using a consensus process with several experienced clinicians, and according to DSM-5 criteria, endorsed by information from the following standardized instruments: Kiddie Schedule for Affective Disorders and Schizophrenia (K-SADS^25^), Diagnostic Interview for ADHD in Adults (DIVA 2.0^26^), Autism Diagnostic Observation Schedule 2nd Edition (ADOS-2^27^), and Autism Diagnostic Interview- Revised (ADI-R^28^). IQ testing was performed with the following measures depending on participant’s age and verbal capacities: Wechsler Intelligence Scale for Children-IV (WISC-IV^29^), Wechsler Adult Intelligence Scale-IV (WAIS-IV^30^), and the Leiter International Performance Scale-Revised^30^).

### Laboratory analysis

Our approach to laser ablation-inductively coupled plasma-mass spectrometry (LA-ICP-MS) tooth metals analysis and assigning developmental times has been detailed elsewhere^31,32^. Briefly, teeth are sectioned vertically along the labio-lingual/buccal-lingual plane and sampled parallel to the dentine-enamel junction from the dentine horn tip towards the tooth cervix. Depending on scan length, each tooth is sampled at 152 locations on average. Temporal information is assigned to sampling points using the neonatal line, a histological feature formed in enamel and dentine at the time of birth, and additional pre- and postnatal incremental markings. A New Wave Research NWR-193 (ESI, USA) laser ablation unit equipped with a 193 nm ArF excimer laser was connected to an Agilent Technologies 8800 triple-quad ICP-MS (Agilent Technologies, USA). Ablation was carried out under a helium atmosphere which is mixed with argon via Y-piece before introduction to the ICP-MS. Instrument sensitivity (maximum analyte ion counts), oxide formation (^232^Th^16^O^+^/^232^Th^+^, <0.3%) and fractionation (^232^Th^+^/^238^U^+^, 100 ± 5%) were monitored daily using NIST SRM 612 (trace elements in glass). Data were collected as metal to calcium ratios (e.g., ^208^Pb:^43^Ca) to control for any variations in the mineral content within a tooth and between samples.

### Recurrence quantification analysis

**Fig. 1:**
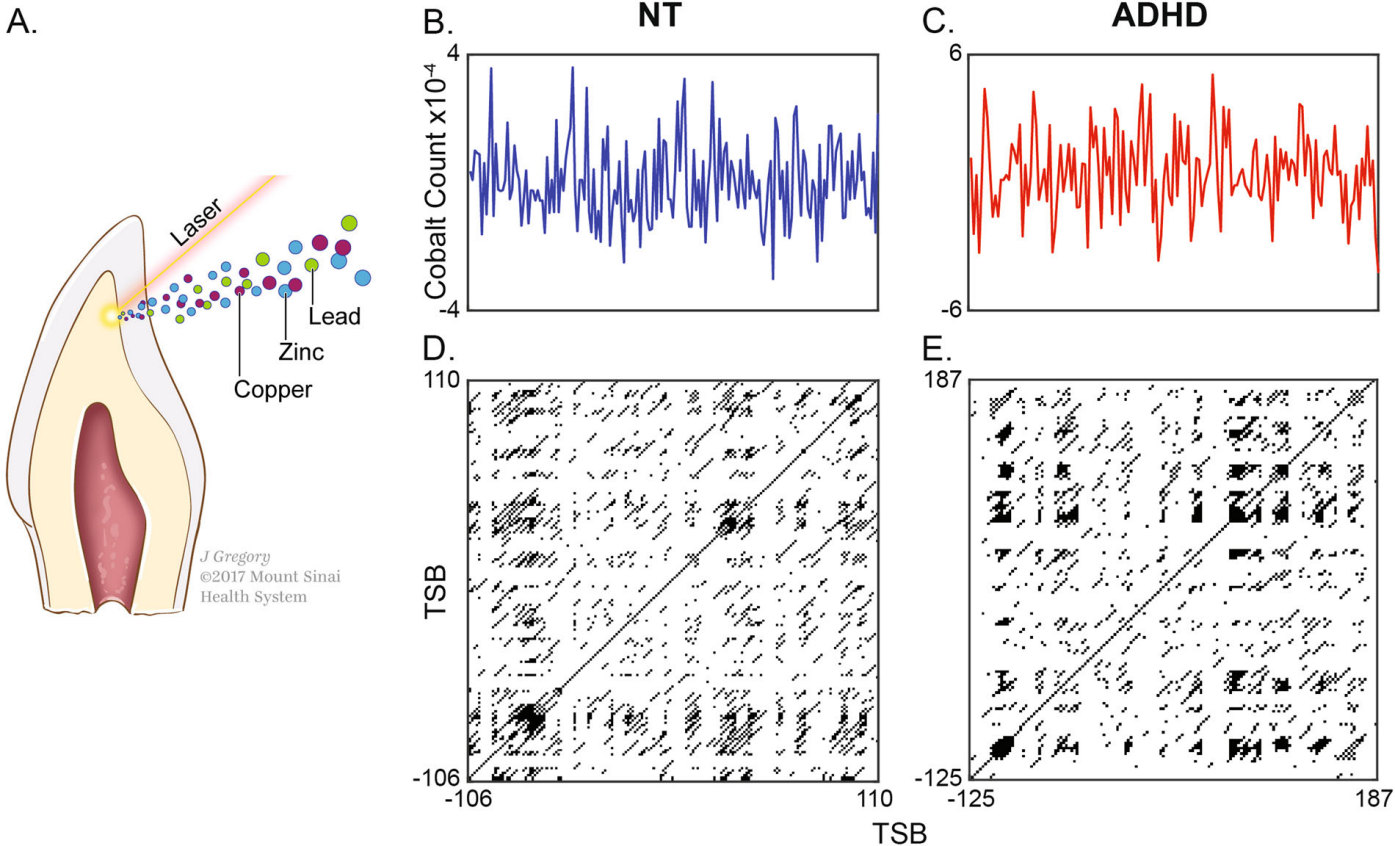
Overview of study design. **a** Collected deciduous teeth were analyzed using laser ablation-inductively coupled plasma-mass spectrometry to generate temporal profiles of metal uptake during fetal and postnatal development. **b** Example cobalt exposure profile in a typically-developed (TD) control subject ranging from −106 to 110 days since birth. **c** Example cobalt exposure profile in a subject diagnosed with ADHD ranging from −125 to 187 days since birth. **d** Recurrence plot generated from control element trace in panel **b**. This graphical analytical tool presents cycIical processes as diagonal lines; in recurrence quantification analysis (RQA), the duration (mean diagonal line length, MDL), complexity (entropy), and determinism (proportion of diagonal elements) of cyclical processes are analyzed to characterize dynamical features in elemental time series. **e** Recurrence plot generated from ADHD case in panel **c**. The relative abundance of laminar states (black structures) and attenuated diagnoal length indicate reduced periodicity

As previously described in Curtin et al.^19,33^, we used non-linear methods—recurrence quantification analysis (RQA) and cross-recurrence quantification analysis (CRQA)—to characterize dynamic, cyclical properties of environmental exposures and their metabolism. Briefly, time-series data sampled from teeth were used to construct recurrence plots of single elements or cross-recurrence plots of two elements. We investigated the temporal features generated by recurrence plots, focusing on determinism, mean diagonal length, entropy, and recurrence time. These features measure diagonal line structures, or cyclical events, in recurrence plots. Determinism measures the ratio of diagonal lines (cyclical events) to vertical and/or horizontal lines (non-cyclical events) effectively defining the periodicity of an elemental time-series. Mean diagonal length measures the mean duration of diagonal lines or cycles, and recurrence time measures the interval between these cycles. Entropy captures the complexity of cyclic activity by measuring the variability in cycle lengths. These measures were similarly generated for CRQA, but captured the temporal relationship between two elemental signals. These methods are summarized in Fig. 1 and were processed with the Cross-Recurrence Toolbox v5.16^34^ in Matlab v2016b (Mathworks).

### Statistical methods

Prior to applying inferential statistical testing, all variables were evaluated to confirm assumptions of normality in value distributions. Values of ±2 SDs were excluded from some variables to meet assumptions of normality. For analyses of recurrence features (RQA/CRQA, described above), linear mixed models were used to test main dichotomous effects of the presence/absence of an ADHD-diagnosis on these features. Twin pairs were modeled as random variables, while also controlling for sex, gestational days, birth weight, and comorbid ASD status. Additional covariates, such as zygosity and IQ, were initially included in model construction but were ultimately excluded as these yielded no improvement in model fit (Akaike Information Criteria, AIC), had no significant effects, or caused no changes in the significance of other covariates included in modeling. False discovery rate (FDR) adjustments, stratified by metal pathway (or cross recurrence), were applied to raw p-values relating to ADHD effects; unless otherwise noted, all reported p-values reflect FDR adjustment. Adjusted p-values less than or equal to 0.05 are reported as significant.

We additionally implemented dimensionality-reduction techniques to investigate the utility of these methods in characterizing neurotypical, ADHD, ASD, and ADHD/ASD comorbid phenotypes. We first applied principal component analysis (PCA), an unsupervised dimensionality-reduction technique, to investigate relationships among features derived from RQA, and evaluate the efficacy of derived components. Measures derived from RQA of single elements and cross-recurrence (CRQA) analyses, including Determinism, Entropy, Mean Diagonal Length, and Recurrence Time, were centered and scaled for PCA. Following inspection of a scree plot, 15 components were retained for subsequent analyses, as these explained >80% of total variance and all estimated eigenvalues were greater than 1. Linear models were used to evaluate if derived components differed between subjects diagnosed as neurotypical, ASD, ADHD, or comorbid with ASD and ADHD. PCA was implemented in R v 3.5.2 with the *factoextra* package.

We next complemented the unsupervised analysis described above with a linear discriminant analysis (LDA), a supervised dimensionality-reduction technique, to evaluate the separation of ADHD, ASD, comorbid ASD/ADHD and neurotypical behavioral phenotypes on the basis of elemental metabolic features extracted via RQA. This analysis simultaneously evaluated all 60 features derived from eight elemental pathways and seven cross-recurrences to separate behavioral diagnoses along a lower dimensional space. Subjects with missing values for any of these features were excluded from this analysis. For each discriminant axis calculated, the correlation between raw RQA features and discriminant scores was calculated to provide a standardized measure of variable loading/importance as it related to a given axis. Analyses were implemented in R v 3.5.2 with the *mass* package.

## Results

### Dysregulation of elemental profiles in ADHD

We first analyzed the cyclical processes of individual elemental profiles in ADHD cases and compared those to neurotypical controls. We found that ADHD cases consistently showed a decrease in overall rhythmicity of metal levels over time, which is measured by *determinsim*; these effects are summarized per-metal in Supplemental Table 2. In ADHD cases, determinism was reduced in the elements cobalt (β = −0.03, *P* = 0.017), lead (β = −0.03, *P* = 0.016), and vanadium (β = −0.03, *P* = 0.01); and, there was a tendency for copper to be reduced (β = −0.03, *P* = 0.054). These results indicate that in ADHD cases there is instability in the cyclic metabolic activity of these elements, relative to what occurs in typically developing twins. We found a significant effect of birth weight in zinc (β = −0.00002, *P* = 0.03), but no other significant effects relating to determinism across metals in our covariate adjustments (i.e., sex, gestational days, birth weight, and ASD).

We also saw significant reduction in the duration and complexity of some elemental cycles. In ADHD cases, the duration of elemental cycles, measured by mean diagonal length, was reduced in lead (β = −0.38, *P* = 0.016) and vanadium (β = −0.25, *P* = 0.01), compared to neurotypical controls (Supplemental Table 2). Furthermore, entropy (a measure of the variability in cycle lengths) was decreased in cobalt (β = −0.13, *P* = 0.017), lead (β = −0.18, *P* = 0.016), and vanadium (β = −0.15, *P* = 0.008) in ADHD cases (Supplemental Table 2). Again, we found birth weight had a significant effect on zinc entropy (β = −0.0001, *P* = 0.03), but none of the other covariates included in these models had significant effects across elements. Additionally, we found no significant differences in the intervals between cyclical processes (i.e., recurrence time) between cases and controls.

### Dysregulation of cross-elemental profiles in ADHD

Next we tested co-regulation of zinc and other elements, because zinc is known to be a central regulator of other metals and its associated pathways have been implicated in protection against toxicants, e.g., lead^35^, and we previously found zinc dysregulation was related to ASD^19^. Decreased determinism was not a characteristic feature of zinc-element interactions in ADHD cases, with the exception of zinc-copper (β = −0.03, *P* = 0.024) (Supplemental Table 3).

The duration of periodic cycles in ADHD cases was disrupted in three out of seven zinc-element profiles. However, after FDR correction, only one of these pathways was significantly different between ADHD cases and neurotypical controls. Mean diagonal length was significantly shorter in zinc-vanadium cross recurrence (β = −0.24, *P* = 0.036), and tended to be reduced in zinc-tin cycles (β = −0.33, *P* = 0.058) and zinc-calcium cycles (β = −0.23, FDR-adjusted *P* = 0.15, raw *p* = 0.04) (Supplemental Table 3). Entropy was also disrupted in ADHD cases in zinc-vanadium (β = −0.11, *P* = 0.036) (Supplemental Table 3). Zinc-vanadium (β = −0.71, *P* = 0.036) was the only element-pair to show differences in recurrence time, reflecting the interval durations between cyclic periods (Supplemental Table 3).

### Phenotypic signatures of ADHD, ASD, ADHD/ASD combined vs neurotypical cases

We have previously reported how elemental regulation in ASD differs from typical development^19,24^. While the analyses described thus far focused on contrasting individuals with ADHD from neurotypical controls while controlling for ASD, here we sought to leverage the high-dimensional features derived from multiple pathways to distinguish signatures unique to these diagnoses. We applied principal component analysis (PCA) and linear discriminant analysis (LDA) to leverage both unsupervised and supervised dimensionality-reduction techniques to achieve this goal.

**Fig. 2:**
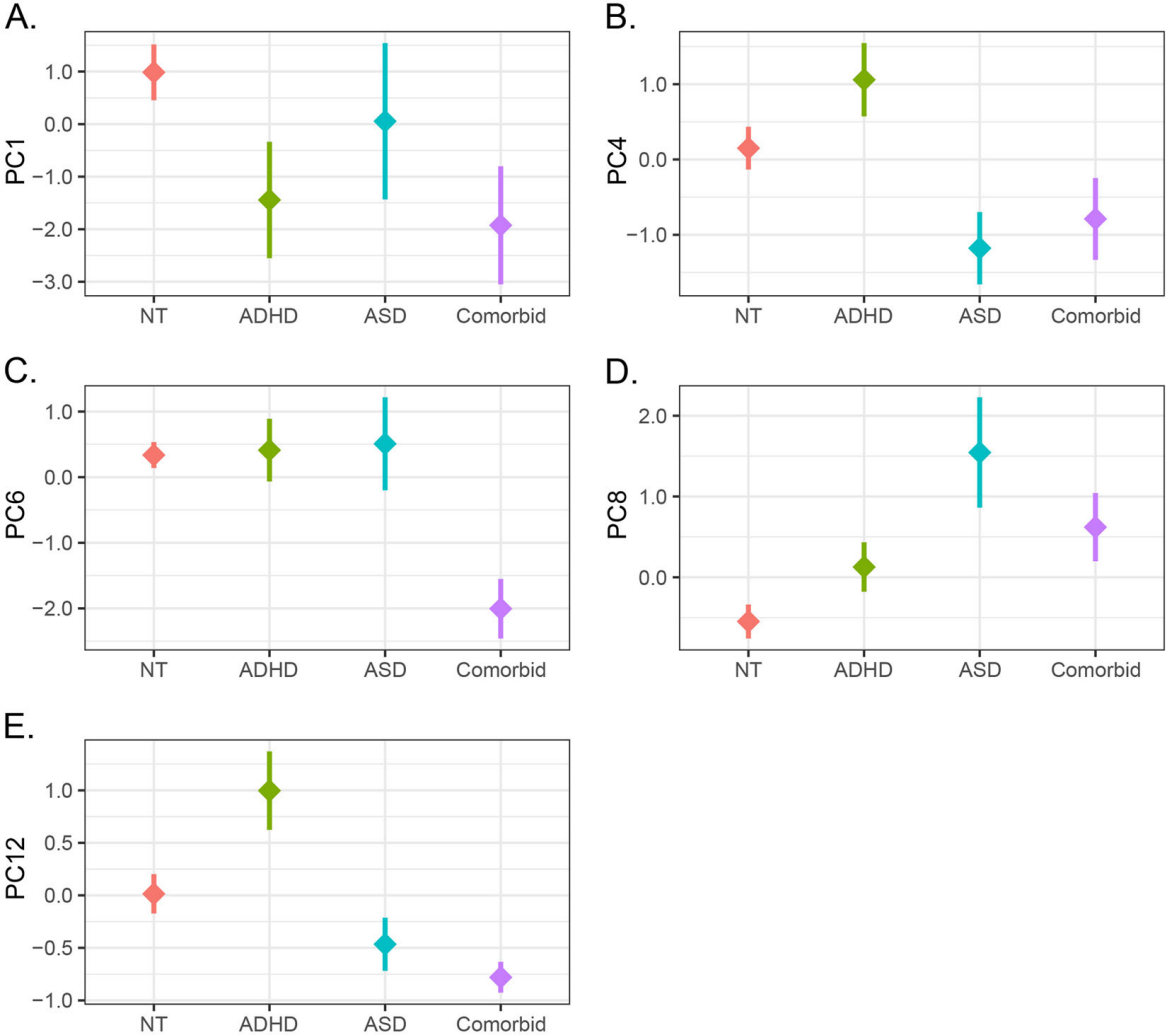
Principal components underlying elemental metabolism in ASD and ADHD. Panels show mean ± SEM for component scores along principal components 1, 4, 6, 8, and 12

Our initial analysis with PCA identified 15 components with eigenvalues exceeding 1, accounting for 82.79% of total variance across RQA/CRQA features. Among this subset of components, we compared component scores among participants, and identified 5 axes relating to neurobehavioral phenotypes, as shown in Fig. 2. Along the first principal component, we identified a significant reduction in component scores relative to neurotypical controls both for ADHD subjects (β = −2.42, *P* = 0.04) and comorbid cases (β = −2.91, *P* = 0.02), as shown in Fig. 2 (panel A). We also identified factors relevant to ADHD along the 12^th^ principal component, which was significant elevated in ADHD participants (β = 0.98, *P* = 0.006), but was depressed in subjects comorbid for ASD/ADHD (β = −0.79, *P* = 0.03). In principal components 4 and 8, we found effects associated with ASD diagnosis, with significantly reduced scores on principal component 4 (β = −1.33, *P* = 0.04), and elevated scores on component 8 for ASD (β = 2.09, *P* < 0.0001) and comorbid cases (β = 1.19, *P* = 0.01). Dysregulation along principal component 6 was specific to comorbid ASD/ADHD cases, with significant reductions relative to neurotypical controls (β = −2.34, *P* < 0.0001). Supplementary Fig. 1 provides the variable loadings associated with each component.

**Fig. 3:**
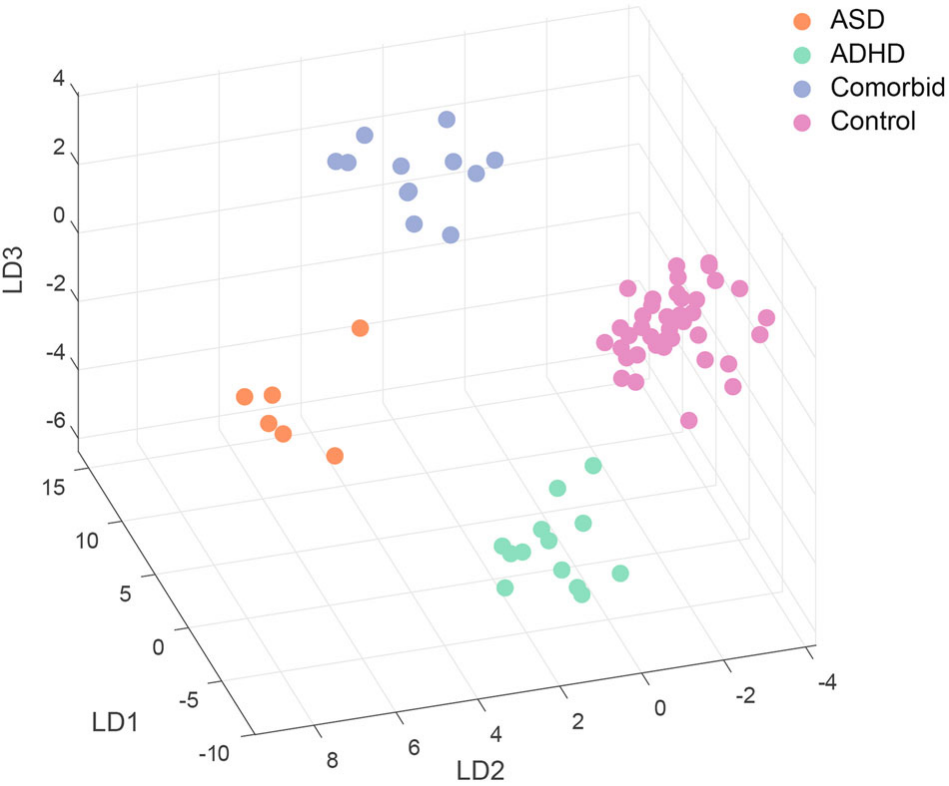
ASD and ADHD-related dimensions in feature-space. Symbols show projection of each subject, identified as a typically developing control or ADHD, ASD, or ADHD/ASD comorbid case, on a subspace derived from linear discriminant analysis (LDA). X-, Y-, and Z-axes shows scores on the first (LD1), second (LD2), and third (LD3) dimensions derived from the LDA procedure

**Fig. 4:**
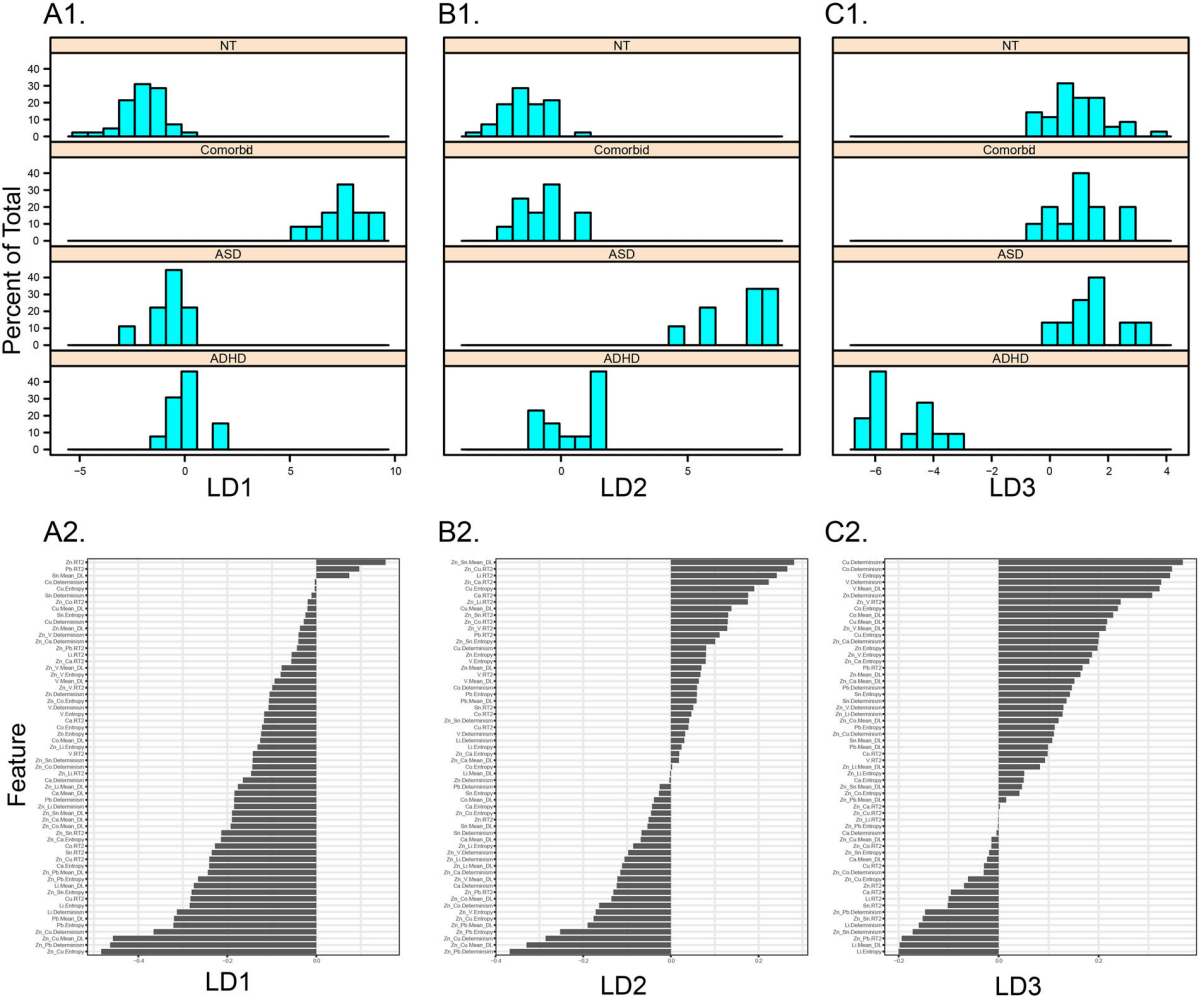
Unique metabolic signatures of neurodevelopmental phenotypes. **A1, B1, C1** show density profiles for neurotypical, ADHD, ASD, and ADHD/ASD comorbid cases along linear discriminant (LD) axes 1, 2, and 3, respectively, with LD1 primarily separating comorbid cases, LD2 distinguishing ASD and ADHD cases, and LD3 separating ADHD cases from other phenotypes. **A2**, **B2**, **C2** show standardized loadings, i.e., correlations between RQA/CRQA features and LD1, LD2, and LD3, respectively

We complemented this approach with a linear discriminant analysis (LDA) to visualize separation of these classes across a low-dimensional feature space. This projection, shown in Fig. 3, emphasizes the separation of neurodevelopmental phenotypes by elemental metabolic features. In Fig. 4, we show how the separation of each neurodevelopmental phenotype manifests across linear discriminant axes (A1, B1, C1), and the standardized loadings of RQA features on these dimensions (A2, B2, C2). The first axis, LD1, primarily captures the separation of comorbid ADHD/ASD cases from neurotypical, ASD, and ADHD cases (Fig. 4, A1), with zinc-copper cycle complexity (entropy) and duration (mean diagonal length), and zinc-lead determinism (rhythmicity) contributing most to this separation (Fig. 4, A2). Similarly, LD2 primarily captures the separation of ASD and ADHD cases (Fig. 4, B1), though along this axis zinc-tin mean diagonal length, zinc-copper recurrence time, and lithium recurrence time also contribute to this separation, in addition to the features described above. LD3 captures the separation of ADHD cases from all other phenotypes (Fig. 4, C1), primarily driven by copper and cobalt determinism, vanadium entropy and determinism, and lithium entropy and mean diagonal length, among other features. The integration of dynamical features extracted from multiple elemental pathways thus identifies a signature associated with each behavioral phenotype. The complete listings of the relative contribution of each feature to each discriminative axis are provided in Fig. 4 (A2, B2, C2).

## Discussion

Our results show that cyclical processes involved in the metabolism of essential elements and toxic metals during fetal and early postnatal development differ significantly in ADHD and neurotypical development. We used tooth matrix biomarkers to measure detailed pre- and postnatal temporal profiles of lead, cobalt, zinc, vanadium, and other elements, and tested the hypothesis that alterations of metal metabolism cycles are associated with ADHD. Overall, we found that the stability, duration, and complexity of cyclical processes were reduced in ADHD cases compared to controls. These findings jointly suggest that the metabolism of essential and toxic elements is affected in ADHD. Critically, we were able to identify distinct cyclical properties specific to ADHD alone, as well as shared elemental pathways common to both ADHD and ASD.

In ADHD, we saw decreased cycle stability (determinism), duration (mean diagonal length), and complexity (entropy) in cobalt, copper, lead, zinc, and vanadium exposure profiles. Previous studies have shown that increased levels of lead are correlated with the severity of ADHD symptoms^7^, specifically, rather than neurodevelopmental conditions like ASD in general. Interestingly, we were not only able to identify distinct elemental signatures for those with either ADHD or ASD, but also in combined behavioral presentations. This finding at the molecular level is in broad agreement with clinical features of these conditions where significant overlap is observed in symptomatology^36–38^.

Our findings are also bolstered by human and animal studies where exposure to environmental chemicals altered pathways relevant to the symptoms observed in ADHD^39–41^. A handful of studies describe the possible roles for zinc in ADHD. Zinc is an essential element involved in dopamine pathways that are assumed to be involved in ADHD etiology. Zinc deficiency has been hypothesized to cause dysfunction of the dopamine transporter^42^, impaired dopaminergic transmission^41^ and modulation of melatonin, a regulator of dopamine function and potential pathway of amphetamine treatment of ADHD^43^ or ADHD-related sleep problems^44^. Several enzymes believed to have an essential role in the neurophysiology of ADHD are dependent on copper^45^. A dysregulation of zinc or copper may also increase susceptibility to oxidative damage of tissues or oxidative stress of the brain by damaging antioxidant defenses, a possible pathophysiology of ADHD^41,46,47^. Excess copper may promote the oxidation of dopamine and its metabolite salsolinal, leading to the degeneration of dopaminergic neurons^46^. Lead may contribute to dopaminergic dysfunction^48^ and disrupt the blood-brain barrier^49^.

Our study is limited by a small sample size, although it was sufficient to distinguish significant differences in cyclical metal properties between ADHD, ASD, ADHD/ASD combined, and neurotypical development. As a consequence of this, it was not possible to implement robust cross-validation procedures, and these results should therefore not be interpreted as a generalizable algorithm for predictive phenotyping or diagnostic assessment. Nonetheless, the utility of both supervised and unsupervised methods in separating neurodevelopmental phenotypes, and the general overlap in features that discriminate ASD & ADHD, suggest that elemental dynamics, particularly relating to zinc periodicity, may provide a powerful signature for future studies to leverage for predictive phenotyping. The differentiation of behavioral phenotypes including ADHD, ASD and their combined presentation from elemental metabolic features further emphasizes the significance of these processes in neurodevelopment, and the potential future applications of these methods in a broader sample. The use of twins in our study allowed us to control for underlying genetic factors and to increase our power to detect differences between diagnostic groups. However, further work is needed in non-twin participants to confirm and extend our results. Organic environmental chemicals may also play a role in ADHD, a topic not addressed in our study.

Studies of environmental exposures and ADHD have primarily focused on the magnitude of exposures as determined by concentrations in blood, urine or other biological matrices. By analyzing dynamical processes in elemental exposure profiles, we have uncovered differences in elemental metabolism that would not be apparent in measures of exposure intensity alone. Furthermore, our findings have implications for early detection of ADHD, ASD, and ADHD/ASD combined cases because the signatures we have observed are present prenatally and in early postnatal development. Pathways we have identified, once validated, may also offer targets for prevention of toxic exposures and early intervention in the form of modification of elemental metabolism towards a healthy molecular phenotype.

## Supplementary information

Supplemental Material
