# Supplementary material for: Dynamical properties of elemental metabolism distinguish attention deficit hyperactivity disorder from autism spectrum disorder
Source: Transl Psychiatry. 2019 Sep 25;9:238. doi: 10.1038/s41398-019-0567-6 (PMC6760156; doi:10.1038/s41398-019-0567-6)
Supplement: Supplementary file 1 — Supplemental Material [file 41398_2019_567_MOESM1_ESM.docx]

**Supplemental Material for:**

**Dynamical properties of elemental metabolism distinguish attention deficit hyperactivity disorder from autism spectrum disorder**

Emergent Dynamical Systems (EDS) group, Kristiina Tammimies, Johan Isaksson, Charlotte Willfors, Sven Bölte

E.D.S. group (all authors contributed equally): Austin, C., Curtin, P., Curtin, A., Gennings, C., Arora, M.


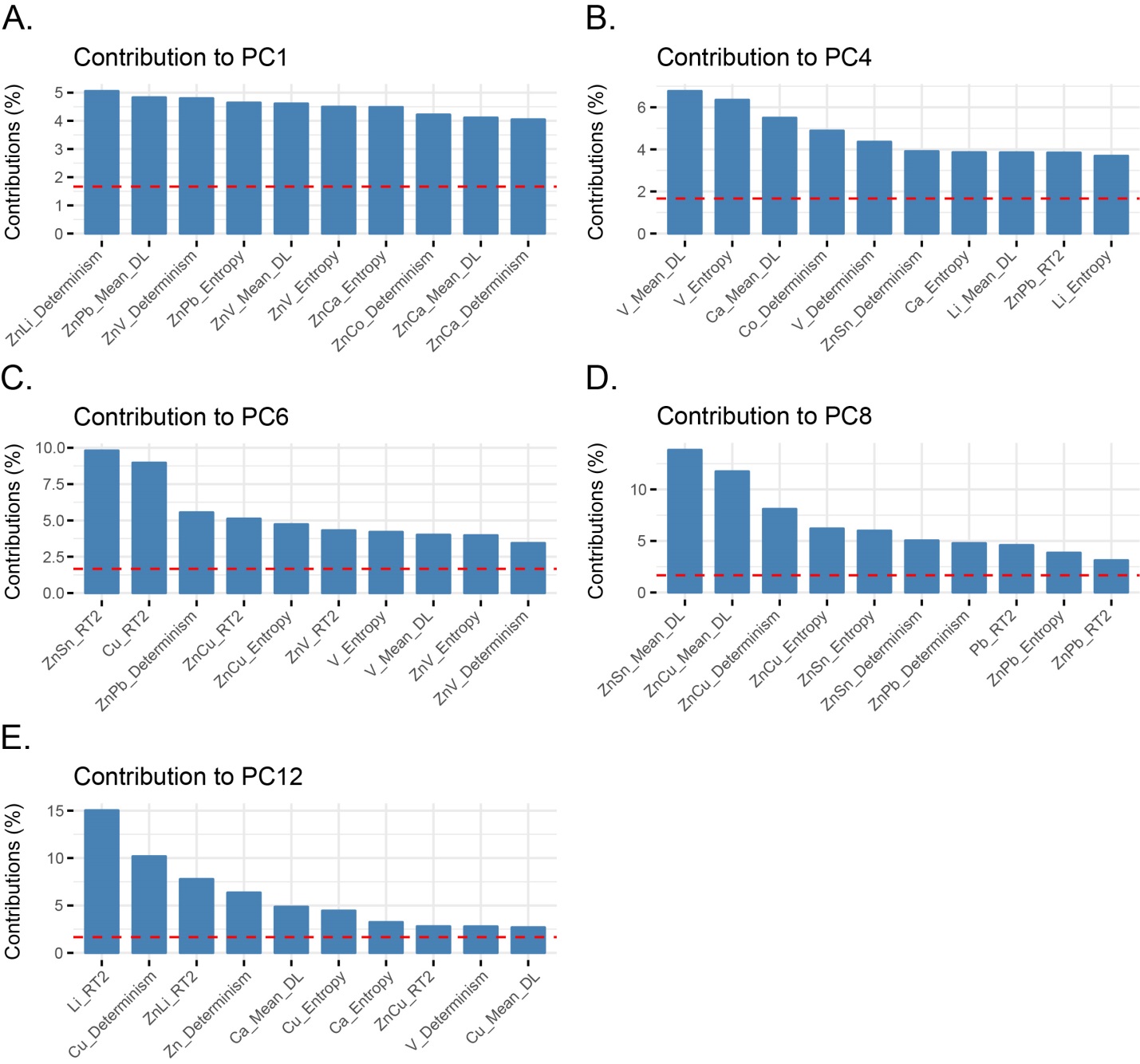


**Supplemental Figure 1.** Variable loadings on principal components associated with neurobehavioral phenotypes. Plots show standardized variable loadings, reflecting the relative weighted contribution of each variable to a given principal component, for the 10 variables most strongly associated with a given component. Dashed red line shows the contribution expected by chance.

**Supplemental Table 2. Results of single-recurrence analyses**

| **Measure** | **Metal** | **β** | **Lower Bound** | **Upper Bound** | **P** | **FDR** |
| --- | --- | --- | --- | --- | --- | --- |
| **Determinism** | Ca | 0.00 | -0.02 | 0.02 | 0.793 | 0.793 |
|  | Co | -0.03 | -0.05 | -0.01 | **0.008** | **0.017** |
|  | Cu | -0.03 | -0.06 | -0.01 | **0.014** | 0.054 |
|  | Li | -0.01 | -0.05 | 0.03 | 0.621 | 0.621 |
|  | Pb | -0.03 | -0.05 | -0.01 | **0.012** | **0.016** |
|  | Sn | -0.01 | -0.03 | 0.02 | 0.524 | 0.524 |
|  | V | -0.03 | -0.05 | -0.01 | **0.008** | **0.010** |
|  | Zn | -0.02 | -0.04 | 0.00 | 0.075 | 0.115 |
| **Entropy** | Ca | -0.12 | -0.23 | 0.00 | 0.052 | 0.206 |
|  | Co | -0.13 | -0.22 | -0.04 | **0.009** | **0.017** |
|  | Cu | -0.12 | -0.24 | 0.01 | 0.076 | 0.101 |
|  | Li | 0.03 | -0.09 | 0.15 | 0.581 | 0.621 |
|  | Pb | -0.18 | -0.30 | -0.06 | **0.005** | **0.016** |
|  | Sn | -0.08 | -0.19 | 0.03 | 0.132 | 0.264 |
|  | V | -0.15 | -0.24 | -0.06 | **0.002** | **0.008** |
|  | Zn | -0.10 | -0.20 | 0.00 | **0.049** | 0.115 |
| **Mean Diagonal Length** | Ca | -0.21 | -0.53 | 0.10 | 0.173 | 0.339 |
|  | Co | -0.17 | -0.35 | 0.01 | 0.058 | 0.070 |
|  | Cu | -0.31 | -0.64 | 0.03 | 0.073 | 0.101 |
|  | Li | 0.10 | -0.09 | 0.29 | 0.292 | 0.621 |
|  | Pb | -0.38 | -0.68 | -0.09 | **0.012** | **0.016** |
|  | Sn | -0.11 | -0.35 | 0.13 | 0.357 | 0.476 |
|  | V | -0.25 | -0.42 | -0.07 | **0.007** | **0.010** |
|  | Zn | -0.23 | -0.50 | 0.04 | 0.087 | 0.115 |
| **Recurrence Time** | Ca | -0.46 | -1.28 | 0.36 | 0.254 | 0.339 |
|  | Co | -0.32 | -0.66 | 0.03 | 0.070 | 0.070 |
|  | Cu | -0.19 | -1.16 | 0.77 | 0.682 | 0.682 |
|  | Li | -0.15 | -0.55 | 0.25 | 0.448 | 0.621 |
|  | Pb | -0.63 | -1.32 | 0.05 | 0.068 | 0.068 |
|  | Sn | -0.46 | -0.94 | 0.03 | 0.064 | 0.257 |
|  | V | -0.31 | -0.71 | 0.09 | 0.118 | 0.118 |
|  | Zn | 0.29 | -0.86 | 1.45 | 0.605 | 0.605 |

Beta estimates reflect comparison of participants with ADHD diagnosis to those without, i.e. negative values imply a smaller mean in ADHD cases. Lower and Upper Bounds reflect 95% confidence intervals on parameter estimates.

**Supplemental Table 3. Results of cross-recurrence analyses**

| **Measure** | **Metal** | **β** | **Lower Bound** | **Upper Bound** | **P** | **FDR** |
| --- | --- | --- | --- | --- | --- | --- |
| Determinism | ZnCa | -0.01 | -0.03 | 0.02 | 0.674 | 0.674 |
|  | ZnCo | 0.00 | -0.02 | 0.03 | 0.721 | 0.721 |
|  | ZnCu | -0.03 | -0.05 | -0.01 | **0.006** | **0.024** |
|  | ZnLi | -0.02 | -0.04 | 0.01 | 0.201 | 0.280 |
|  | ZnPb | -0.02 | -0.05 | 0.01 | 0.113 | 0.374 |
|  | ZnSn | -0.01 | -0.04 | 0.03 | 0.668 | 0.668 |
|  | ZnV | -0.01 | -0.04 | 0.01 | 0.249 | 0.248 |
| Entropy | ZnCa | -0.09 | -0.19 | 0.01 | 0.075 | 0.150 |
|  | ZnCo | -0.02 | -0.13 | 0.08 | 0.631 | 0.721 |
|  | ZnCu | -0.07 | -0.16 | 0.02 | 0.103 | 0.137 |
|  | ZnLi | -0.07 | -0.18 | 0.04 | 0.198 | 0.280 |
|  | ZnPb | -0.07 | -0.16 | 0.03 | 0.187 | 0.374 |
|  | ZnSn | -0.12 | -0.25 | 0.01 | 0.065 | 0.129 |
|  | ZnV | -0.11 | -0.21 | -0.01 | **0.027** | **0.036** |
| Mean Diagonal Length | ZnCa | -0.23 | -0.46 | 0.00 | **0.046** | 0.150 |
|  | ZnCo | -0.06 | -0.31 | 0.19 | 0.639 | 0.721 |
|  | ZnCu | -0.19 | -0.42 | 0.03 | 0.087 | 0.137 |
|  | ZnLi | -0.15 | -0.39 | 0.09 | 0.210 | 0.280 |
|  | ZnPb | -0.06 | -0.33 | 0.21 | 0.650 | 0.650 |
|  | ZnSn | -0.33 | -0.60 | -0.07 | **0.015** | 0.058 |
|  | ZnV | -0.24 | -0.45 | -0.03 | **0.027** | **0.036** |
| Recurrence Time | ZnCa | -0.67 | -1.52 | 0.18 | 0.118 | 0.157 |
|  | ZnCo | -0.45 | -1.11 | 0.21 | 0.167 | 0.669 |
|  | ZnCu | -0.13 | -1.36 | 1.10 | 0.828 | 0.828 |
|  | ZnLi | -0.27 | -0.92 | 0.38 | 0.403 | 0.403 |
|  | ZnPb | 0.35 | -0.59 | 1.30 | 0.445 | 0.594 |
|  | ZnSn | 0.38 | -0.47 | 1.22 | 0.367 | 0.489 |
|  | ZnV | -0.71 | -1.29 | -0.14 | **0.018** | **0.036** |

Beta estimates reflect comparison of participants with ADHD diagnosis to those without, i.e. negative values imply a smaller mean in ADHD cases. Lower and Upper Bounds reflect 95% confidence intervals on parameter estimates.
